# Applications of Alzheimer’s disease staging to clinical trials

**DOI:** 10.18632/aging.204482

**Published:** 2023-01-05

**Authors:** Joseph Therriault, Serge Gauthier, Pedro Rosa-Neto

**Affiliations:** 1 Translational Neuroimaging Laboratory, The McGill University Research Centre for Studies in Aging, Douglas Hospital, McGill University, Canada; 2 Department of Neurology and Neurosurgery, McGill University, Canada; 3 Montreal Neurological Institute, Canada

**Keywords:** disease staging, Alzheimer’s disease, tau, biomarkers, clinical trials

Staging of disease allows for the estimation of disease severity based on the identification of important points in the natural history of a disease. Crucially, grouping together patients at similar stages of disease severity permits for the enrichment of clinical trials [1]. In Alzheimer’s disease (AD), where a substantial portion of the disease unfolds in the absence of symptoms, disease staging systems can potentially inform the design, screening and recruitment strategies for future AD clinical trials [2]. In this article, we describe potential strategies that disease staging can be applied to AD clinical trials for population enrichment and monitoring target engagement, with an emphasis on preclinical AD.

Individuals without cognitive impairment are highly heterogenous in terms of their risk of future cognitive decline [3]. Even restricting trial enrolment criteria to amyloid-β positive individuals results in highly heterogenous rates of short-term cognitive decline. An alternative is to select and enrol participants who have amyloid-β positivity and either (i) no tau pathology (ii) early tau pathology, i.e., Braak I–II or (iii) intermediate tau pathology (Braak III–IV). These pathologies (and their severity) are difficult to identify based on cognitive assessments, even in highly specialized centres [4]. Biomarkers therefore are needed to identify AD pathologies *in vivo*. Several recent clinical trials screened potential participants prior to enrolment in order to only include individuals who could benefit from a specific therapy. Moreover, the recent phase II donanemab clinical trial also restricted enrolment criteria to intermediate levels of tau pathology as determined by tau-PET. A potential strategy could be to assign amyloid-β positive participants to different clinical trials depending on their severity of tau pathology, evaluated using PET-based Braak staging. Having groups of participants with greater similarity in rates of short-term cognitive decline could increase statistical power for future trials by reducing standard error of the mean for cognitive decline within a group. Using PET for disease staging has two important advantages over fluid biomarkers. The topographical information for PET imaging allows for inferences regarding AD severity in an individual subject. Furthermore, because phosphorylated tau (p-tau) biomarkers in both CSF and plasma are more closely associated with amyloid-β plaques than tau neurofibrillary tangles [5], especially at early disease stages. Therefore, tau-PET will likely be more informative for disease prediction as compared to p-tau biomarkers in CSF or plasma.

Tau-PET may also be useful for monitoring disease modification as a secondary outcome in pivotal randomized controlled trials. While it is not fully understood whether tau tangles themselves are causative of cognitive decline or merely upstream of it, a therapy that is associated with lower rates of tau accumulation in the experimental group could be interpreted to indicate successful disease modification. For example, if amyloid-positive individuals receiving an anti-amyloid monoclonal antibody do not accumulate tau outside of Braak I–II regions while the placebo arm does, this finding could be used to support primary outcome data (presumably differences in cognitive trajectories) as evidence of disease modification. Such a finding would also provide evidence in humans to support numerous animal models of amyloid-β pathology potentiating tau aggregation and spreading.

In conclusion, disease staging systems using PET imaging have the potential to inform the design, enrichment and monitoring of randomized clinical trials in the field of AD. Given the recent success of lecanemab and the renewed interest in investments into the AD field, PET-based disease staging may increase the success of future anti-amyloid monoclonal antibody therapies, or therapies based on other mechanisms. Despite some evidence suggesting that atypical clinical variants of AD largely have tau distributions compatible with the Braak staging system [6], future work is needed before using biomarker-based staging for atypical AD in clinical trials [7, 8]. However, this concern is less salient for trials targeting the presymptomatic phase of AD.
